# The General Practitioner Barium Meal Service

**Published:** 1972-07

**Authors:** C. Johnson, J. B. Penry

**Affiliations:** from the Department of Radiology, Southmead Hospital, Bristol; from the Department of Radiology, Southmead Hospital, Bristol


### The General Practitioner Barium Meal Service by

*C. Johnson, M.B., B.Ch., D.M.R.D., F.F.R.

and

J. B. Penry, M.B., B.Ch., D.M.R.D., F.F.R.

from the Department of Radiology, Southmead Hospital, Bristol

### INTRODUCTION

The radiology departments at Southmead and Ham Green Hospitals, Bristol, have provided a general practitioner service for general work and barium meals for some years. Unfortunately, the pressure of hospital in-patient and out-patient work led to a waiting period of from three to six months for non-urgent general practitioner barium meal requests. The appointment of one of us (C.J.) to an additional consultant post in 1966 provided for an additional two sessions of general practitioner work.

Since then, "Non-urgent" barium meal requests from general practitioners have been carried out within two or three weeks of receipt of the request and "Urgent" cases are examined, as a rule, within the week, that is to say, as quickly as "Urgent" requests from the hospital out-patient department.

This general practitioner barium meal service is now reasonably efficient in terms of waiting lists and closely approximates to that provided to the hospital out-patient department. A prospective study of the incidence of abnormal findings in this group of patients and in patients referred from the out-patient clinics was therefore made. The abnormal findings in in-patients is included but these are clearly selected cases and not strictly comparable with cases referred by general practitioners.

### METHOD

Additional copies of the reports were retained on all in-patient, out-patient and general practitioner barium meals carried out by the authors during the period of the investigation. Only those examinations actively carried out by the authors were included in the series. The radiological diagnosis was accepted as final as confirmation from additional investigations and follow up could not be obtained in the majority of patients.

### RESULTS

The results are shown in Table 1. It will be seen that of 395 barium meals carried out for general practitioners during the period, 148 or 37.4% showed an abnormality of some sort. The corresponding figures for out-patient and in-patient examinations were 52.8% and 64.0% respectively.

Hiatus hernia and/or gastro-oesophageal reflux was by far the commonest abnormality found in each group and the relative incidence of this disorder in each section was also similiar. The second commonest abnormal finding was that of duodenal ulcer and the incidence of this was also roughly similar in the three groups, though somewhat commoner in the hospital cases. Benign gastric ulcers were distinctly more common in hospital cases, particularly in-patients, than in the general practitioner group, and as might be expected, this was also true of malignancies.

### DISCUSSION

The incidence of abnormal findings in the general practitioner cases in the present series is similar to that found by Davidson (1965) whose figure was of 40.8% compared with our 37.4%.

It is, however, at variance with the experience of Pulvertaft who in a personal communication to Lennon (1969) stated that 62.0% of males and 47.0% of females referred for barium meals by general practitioners showed an abnormality. Cook (1966) reporting the experience in the first year of an "Open access" X-ray department found a 44.0% incidence of abnormal findings for all types of examinations. His impression was, however, that this incidence of pathological findings was "falling off". Though no figures are available, our own impression has been that the incidence of abnormal findings has become somewhat less as the waiting time for a barium meal has become shorter. Our experience does not coincide with the finding of Lennon (1969) that "The weight of published evidence shows that patients referred by general practitioners have a higher abnormality rate than those referred by out-patient consultants", at least as far as barium meals are concerned. However, the incidence of abnormal findings does appear to compare fairly favourably with the abnormal findings in out-patients and seems to be sufficient to make the service a worthwhile one.

There have been several publications stressing the need for radiological services to general practitioners

TABLE 1

| | Total No. Examined | Total Abnormal | Percent. Abnormal | Hiatus Hernia | Gastric Ulcer | Duodenal Ulcer | Carc. Stomach | Carc. Oesoph. | Others |
|---|---|---|---|---|---|---|---|---|---|
| General Practitioner | 395 | 148 | 37.4 | 83 | 9 | 35 | 5 | — | 11 |
| Out-Patients | 178 | 94 | 52.8 | 50 | 10 | 22 | 5 | 2 | 11 |
| In-Patients | 139 | 89 | 64.0 | 29 | 12 | 19 | 12 | — | 21 |

*Present address: Department of Radiology, University Hospital of the West Indies, Kingston, Jamaica

(Macaulay 1962, Eimerl 1962, Vickers 1966). The increased demands on radiology departments by hospital referrals, possibly aggravated by the development of more sophisticated and time consuming radiological techniques, probably means that X-ray facilities for general practitioners have become available rather more slowly that some would have wished. As far as our own X-ray department is concerned, general practitioner barium meal referrals do not overload us unduly. Maintenance of the service in its present fairly efficient form does, however, occupy more than two consultant barium sessions per week and this might clearly be prohibitive in a less well staffed department. The Faculty of Radiologists has stated that general practitioners should have open access to diagnostic facilities at the District General Hospital and that a four-fold increase in the use of radiological services by general practitioners may be expected in the next 10 years (Bull, 1972). Maintenance of comprehensive radiological services in smaller hospitals is generally considered to be uneconomic from the point of view both of the equipment needed and radiologist time.

Eimerl (1962) has stated that the presence of open access radiology departments would save patient time and relieve the work load on clinical departments. Our clinical colleagues have been unaware of this in our hospitals.

The extent to which the general practitioner barium meal service is used in this area is extremely variable. Our own impression has been that the vast majority of referrals come from perhaps a dozen or so practices in a city of over half a million population with, in addition, a large rural "draining area'. One practitioner has told us that before the service reached its present form, he frequently referred patients for a consultant medical or surgical opinion largely in order to get a barium meal carried out within a reasonable space of time. On the other hand, another doctor stated that he considered that if a barium examination was indicated, then a clinical consultant opinion was also necessary. Practitioner "awareness" of the service is obviously also a factor in the extent to which a doctor refers patients. Levitt (1964) has referred to this and has stated that where such services are available, practitioners are not using them as fully as they might.

Our findings would indicate that the general practitioner barium meal service is a worthwhile one which may enable the family doctor to continue the management of cases which, though causing considerable morbidity, may not require hospitalisation or a clinical consultant opinion. Provided the service offered is reasonably efficient, it can, in our opinion, serve only to enhance the standard of general practice. Whether the present radiological services of those planned for the immediate future will be able to cope with the demands of such a service is a matter of conjecture.

**REFERENCES**

1. BULL, J. W. D. 1972 Rationalisation of Diagnostic Radiological Services. Pamphlet issued by the Faculty of Radiologists. Hillingdon Press.
2. COOK, P. L. 1966 Experience in the first year of an "Open Access" X-ray Department. British Medical Journal. **ii** 351.
3. DAVIDSON, J. W. 1965 General Practitioner Referrals to a Diagnostic X-ray Department. Journal of The College of General Practitioners, **10,** 51.
4. EIMERL, T. S. 1962 Direct access Diagnostic Facilities in General Practice. Lancet. **i** 851.
5. LENNON, E. A. 1969 How General Practitioners Use X-ray Departments. Health Trends. Vol. i 17.
6. LEVITT, H. N. 1964 Diagnostic Facilities for General Practitioners in England and Wales. Journal of The College of General Practitioners. **8** 312.
7. MACAULAY, H. M. C. 1962 Diagnostic Facilities for General Practitioners. Lancet **i** 791.
8. PULVERTAFT, C. N. Personal communication to E. A. Lennon.
9. VICKERS, A. A. 1966 Letter to The British Medical Journal. Open X-ray Departments. British Medical Journal. **ii** 520.

**ACKNOWLEDGEMENT**

We are grateful to Dr. I. S. Bailey for his helpful criticism of this paper.

